# Acute exposure to diesel particulate matter promotes collective cell migration in thyroid cancer cells

**DOI:** 10.3389/ftox.2023.1294760

**Published:** 2023-11-30

**Authors:** Sheena Yi-Hsin Cheng, Shih-Yuan Huang, Shih-Ping Cheng

**Affiliations:** ^1^ Taipei European School, Taipei, Taiwan; ^2^ Department of Medical Research, MacKay Memorial Hospital, Taipei, Taiwan; ^3^ Department of Surgery, MacKay Memorial Hospital and MacKay Medical College, Taipei, Taiwan; ^4^ Institute of Biomedical Sciences, MacKay Medical College, Taipei, Taiwan

**Keywords:** fine particulate matter, collective cell migration, Wound Healing Assay, air pollution, thyroid cancer

## Abstract

Several ecological studies suggest that ambient air pollution is associated with the occurrence of thyroid cancer. In this study, we used certified diesel particulate matter as a proxy for fine particulate matter. Human thyroid cancer cell lines 8505C and TPC-1 were incubated with different concentrations of NIST1650b for 5 days and subjected to functional assays. We found that NIST1650b treatment did not affect short-term cell growth but reduced colony formation at high concentrations. Notably, NIST1650b-treated cells showed altered morphology toward cluster coalescence following treatment. Wound healing assays revealed that leading-edge cells formed protruding tips while maintaining cell-cell adhesion, and a significantly higher ratio of wound closure following treatment at 10 μg/mL was seen in both cell lines. A weak stimulatory effect on transwell cell migration was observed in 8505C cells. Taken together, our results suggest that fine particulate matter induced a coherent phenotype accompanied by augmented collective cell migration in thyroid cancer cells.

## Introduction

The incidence of thyroid cancer has risen in recent years, including differentiated and undifferentiated thyroid cancer (Cheng et al., 2023). Although the rise is primarily attributed to overdiagnosis, analysis of the Surveillance, Epidemiology, and End Results-9 cancer registry revealed an increase in the incidence and mortality rates for advanced-stage cancer, suggesting a true increase in the occurrence of thyroid cancer (Lim et al., 2017). Furthermore, despite advancements in the detection and treatment of thyroid cancer, mortality rates remain stable (Ratajczak et al., 2021). The pathogenesis of thyroid cancer is largely unclear other than established risk factors such as ionizing radiation and obesity. Lately, flame retardants, particularly polybrominated diphenyl ethers, have been found to be associated with thyroid cancer (Sanabria et al., 2018). More epidemiological studies are needed to identify potential risk factors for thyroid cancer.

We recently explored social and economic factors relating to global thyroid cancer incidence and mortality using the GLOBOCAN 2020 database and found that the mortality-to-incidence ratio was positively correlated with fine particulate matter concentrations among the countries studied (Hsu et al., 2023). Fine particulate matter, or PM_2.5_, which refers to particles ≤ 2.5 μm in aerodynamic diameter, is the major component of ambient air pollutants and commonly results from combustion of fossil and biomass fuels. Among air pollutants, PM_2.5_, particularly submicron particles, is of serious health concern because it contains numerous toxic compounds, penetrates deeper into the lungs, and can infiltrate the alveoli and reach the bloodstream. Studies have shown that PM_2.5_ has a negative impact on multiple organ systems beyond the respiratory system (Garcia et al., 2023). However, the biological consequences of exposure to PM_2.5_ in thyroid cancer are not yet understood. In this study, we aimed to determine changes in tumor phenotype following exposure to fine particulate matter using *in vitro* cellular models.

## Materials and methods

### Cell culture and treatment

Human thyroid cancer cell line 8505C was obtained from the German Collection of Microorganisms and Cell Cultures (DSMZ), Braunschweig, Germany, and TPC-1 from Sigma-Aldrich, St. Louis, MO, United States of America. Both cell lines have been validated as being of thyroid origin and suitable for preclinical studies (Landa et al., 2019). The 8505C cell line was established from a primary undifferentiated thyroid carcinoma resected from a 78-year-old-female (Ito et al., 1993). The TPC-1 cell line was derived from a papillary thyroid cancer that harbors the RET/PTC1 rearrangement (Ishizaka et al., 1989). Cells were cultured in Roswell Park Memorial Institute (RPMI) 1640 medium supplemented with 10% fetal bovine serum (FBS) in a humidified incubator with 5% CO_2_ at 37°C for up to 15 passages.

Diesel particulate matter used in this study was standard reference material certified by the National Institute of Standards and Technology (NIST), Gaithersburg, MD, USA, and was purchased from Sigma-Aldrich (NIST1650b). Based on previous analysis (Don Porto Carero et al., 2001), the NIST1650b suspension contains particles between 40 nm and 2.5 μm and is predominantly composed of polycyclic aromatic hydrocarbons (PAHs) and nito-PAHs. A stock solution (5 mg/mL) of NIST1650b was prepared in dimethyl sulfoxide (DMSO; Sigma-Aldrich) and sonicated for 1 h to avoid agglomeration of the suspension. Prior to experiments, stock preparations were sonicated again to minimize variability in solution composition and then diluted to the required concentrations.

### Cell growth

Cell growth was determined using the Cell Counting Kit-8 (CCK-8) assay (Sigma-Aldrich) as we previously reported with some modifications (Kuo et al., 2023). Following 5-day treatment with different concentrations of NIST1650b, cells were trypsinized, and about 6,000 cells per well were seeded into 96-well plates. Cells were incubated with or without NIST1650b for 24–72 h. The CCK-8 reagent was added to each well and was bioreduced into a formazan product that is soluble in tissue culture media. In our preliminary study, we found that the precipitating of NIST1650b affected absorbance readings to a certain extent. Therefore, following incubation with the CCK-8 reagent for 2 h, the supernatants were transferred to another plate, and absorbance at 450 nm was measured using a microplate reader (Thermo Fisher Scientific, Waltham, MA, USA).

### Cell morphology

Following treatment with different concentrations of NIST1650b for 5 days, cell morphology was evaluated using Diff-Quik staining (Sysmex, Kobe, Japan). In brief, cells were grown on slides until they had a 70%–80% confluence and then fixed with methanol. The slides were dipped in eosinophilic stain solution and basophilic stain solution sequentially. The morphology was observed using a light microscope (Olympus IX71, Japan).

### Clonogenic assay

Following diesel particulate matter treatment for 5 days, the colony-formation assay was performed as previously described (Cheng et al., 2020). About 500 cells per well were seeded into 6-well plates. The maintenance medium contained different concentrations of NIST1650b without replacement for 11 days. Cells were then washed and fixed with a cold mixture of acetic acid and methanol. To visualize colonies, cells were stained with 3% crystal violet dye for 1 h. Colonies with more than 50 cells were manually counted under microscopy.

### Wound healing assay

Cells were seeded into Culture-Insert 3-well dishes (ibidi GmbH, Grafelfing, Germany) following diesel particulate matter treatment for 5 days. When cells reached confluence, the inserts were removed to create two cell-free gaps (Liu et al., 2022). Cells were further incubated with or without NIST1650b for 5 h–20 h. The width of the wound area was photographed, and percentages of wound closure were quantified using the Wound Healing Size Tool plugin (Suarez-Arnedo et al., 2020) of ImageJ software (National Institutes of Health, Bethesda, MD, USA). The plugin applied contrast enhancement through a saturation percentage parameter, a variance filter with adjustable radius, binarization using a threshold value, and hole filling to remove small isolated cell islets. The generated wound area fraction was used as the percentage of wound closures. However, the precipitation from NIST1650b occasionally interfered with the image segmentation algorithm. Prior to segmentation, we applied a custom-built denoising filter to remove dark black NIST1650b particles and proceeded with quantification of wound closure.

### Transwell migration assay

Following 5-day treatment with different concentrations of NIST1650b, about 6,000 cells were trypsinized and resuspended in serum-free media and seeded into transwell inserts (Corning Inc., Corning, NY, USA). The pore size of the transwell inserts was 8 μm. The lower chamber contained complete media containing 10% FBS as a chemoattractant (Kuo et al., 2022). After 24 h, the remaining cells that did not migrate from the top of the membrane were removed using cotton swabs. Cells migrating through the membrane were fixed, stained with Diff-Quik, and counted under a microscope.

### Statistical analysis

Data are presented as the mean ± standard deviation from three independent experiments with two technical replicates each. Differences between groups were analyzed using one-way analysis of variance (ANOVA) followed by Dunnett’s *post hoc* test for multiple comparisons. The data were entered and analyzed using the SPSS version 25 statistical package. A value of *p* < 0.05 was considered statistically significant.

## Results

**FIGURE 1:**
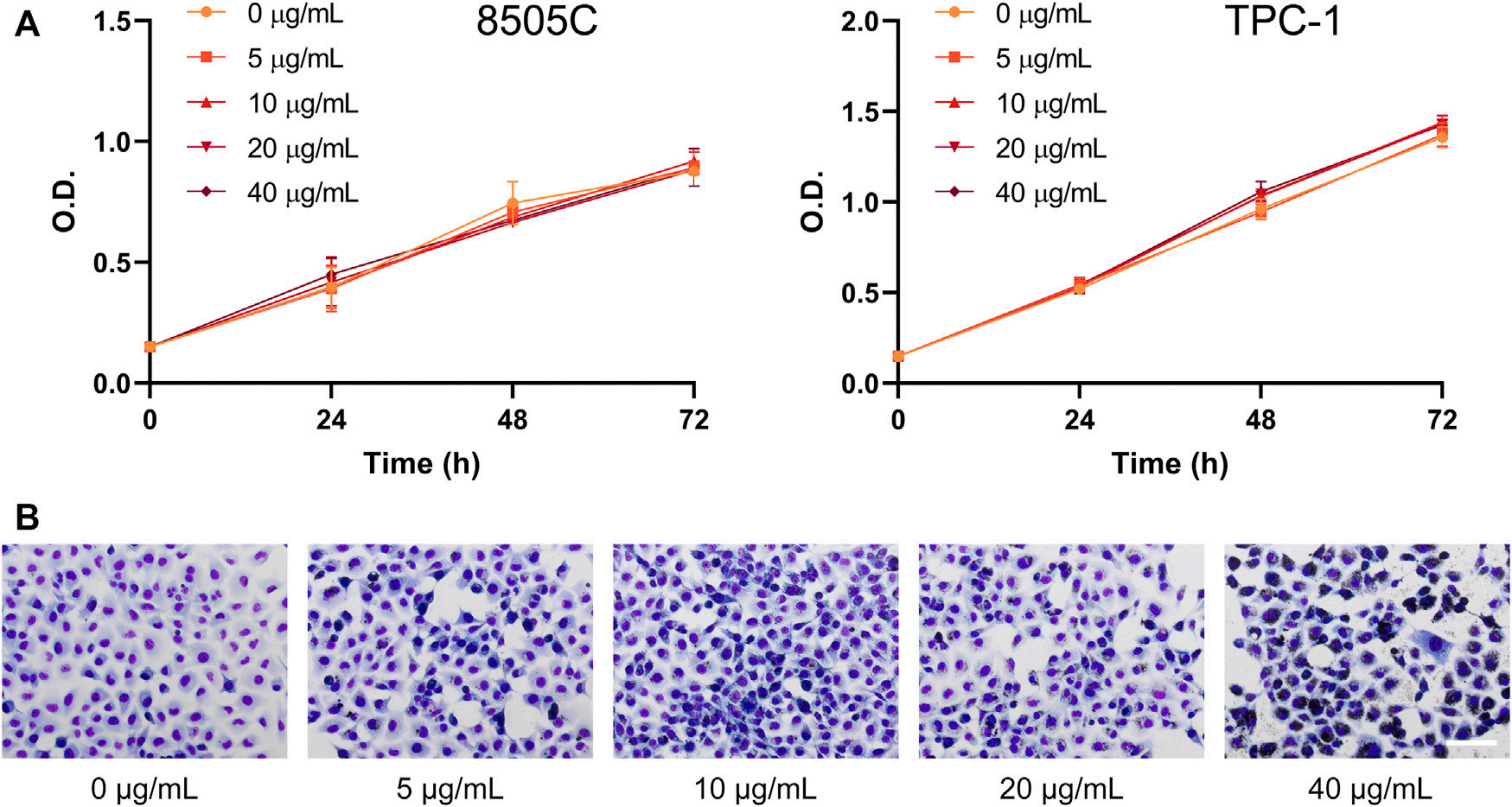
Effects of acute exposure to diesel particulate matter on cell growth and morphology in thyroid cancer cells. Human thyroid cancer cells 8505C and TPC-1 were treated with different concentrations of NIST1650b for 5 days. **(A)** Cell growth was determined using the Cell Counting Kit-8 assay. O. D, optical density. **(B)** Cell morphology of TPC-1 cells was evaluated using Diff-Quik staining. Scale bar, 100 μm.

To evaluate the effects of diesel particulate matter on thyroid cancer cell viability, 8505C and TPC-1 cells were treated with different concentrations of NIST1650b for 5 days, and subsequently cell growth was analyzed for an additional 24–72 h. We used concentrations of 0–40 μg/mL throughout the study based on the results of previous studies (Lee et al., 2020). As shown in Figure 1A, short-term cell growth was not affected by acute exposure to diesel particulate matter. However, thyroid cancer cells exhibited altered morphology. Vehicle control-treated cells were loosely attached to each other, while NIST1650b-treated cells were polygonal and compactly arranged, indicating cluster coalescence (Figure 1B). There were small variations in the nuclear size and shape of NIST1650b-treated cells.

**FIGURE 2:**
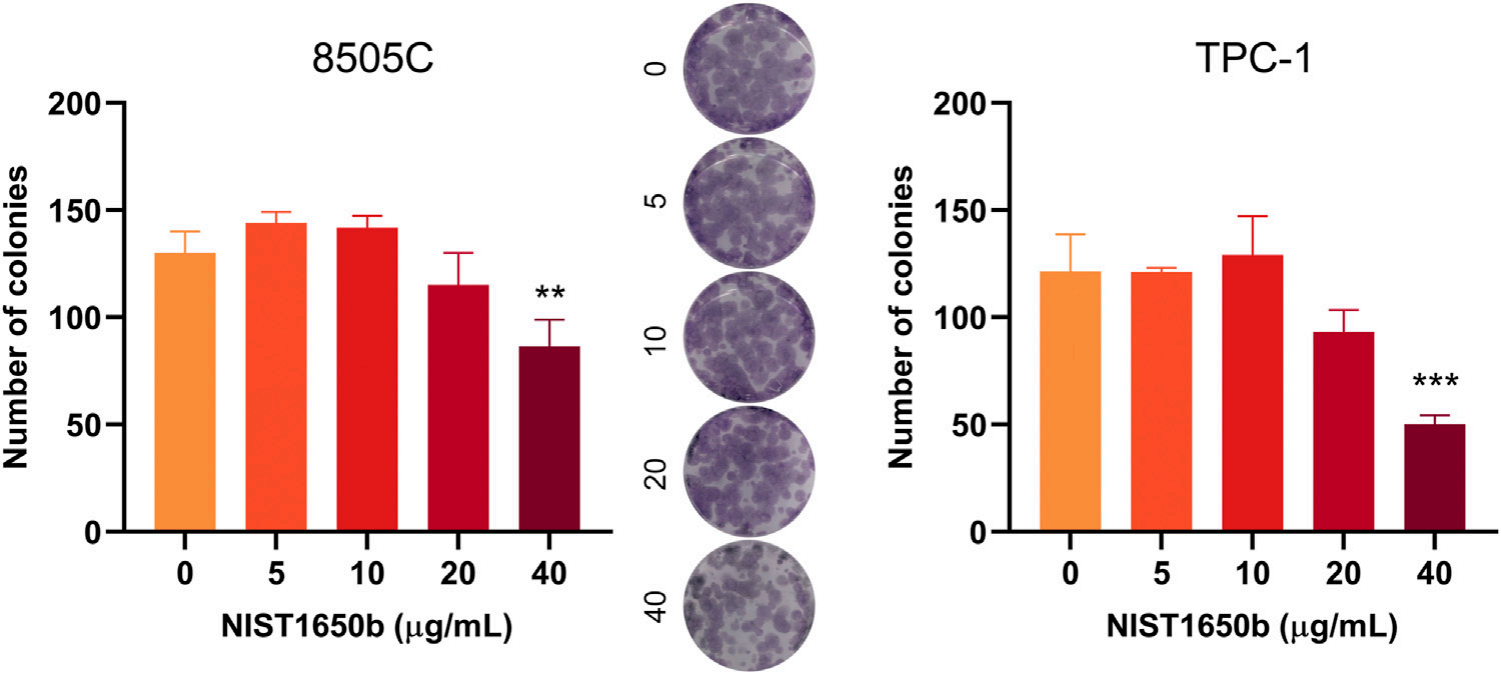
Effects of acute exposure to diesel particulate matter on clonogenicity in thyroid cancer cells. Human thyroid cancer cells 8505C and TPC-1 were treated with different concentrations of NIST1650b for 5 days followed by colony formation assay. Representative photographs of 8505C colonies are shown. ANOVA with Dunnett’s test: **, *p* < 0.01; ***, *p* < 0.001.

We next examined whether exposure to diesel particulate matter affected clonogenicity, the ability of cells to retain their replicative integrity for a longer period of time. There were no significant changes in the number of formed colonies, except a decrease in colony number in thyroid cancer cells treated with 40 μg/mL NIST1650b (Figure 2). This finding suggests that diesel particulate matter interfered with colony formation in thyroid cancer cells only at high concentrations.

**FIGURE 3:**
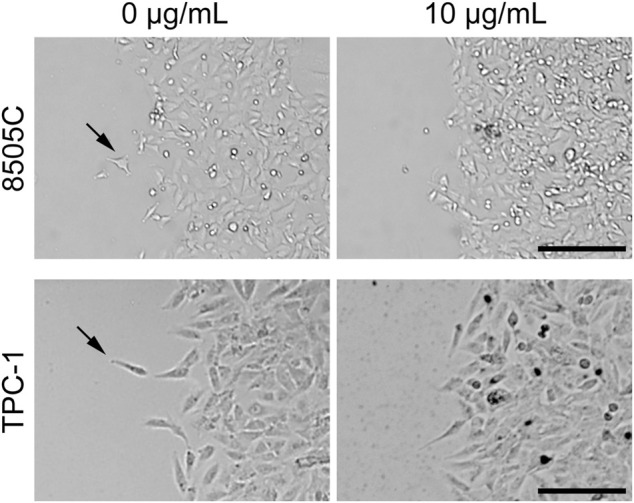
Representative phase-contrast microphotographs of thyroid cancer cells at the leading edge of wound closure following treatment with 10 μg/mL NIST1650b or DMSO vehicle control for 5 days. Arrows indicate cells departing from the leading edge into the wound area. Scale bar, 200 μm.

**FIGURE 4:**
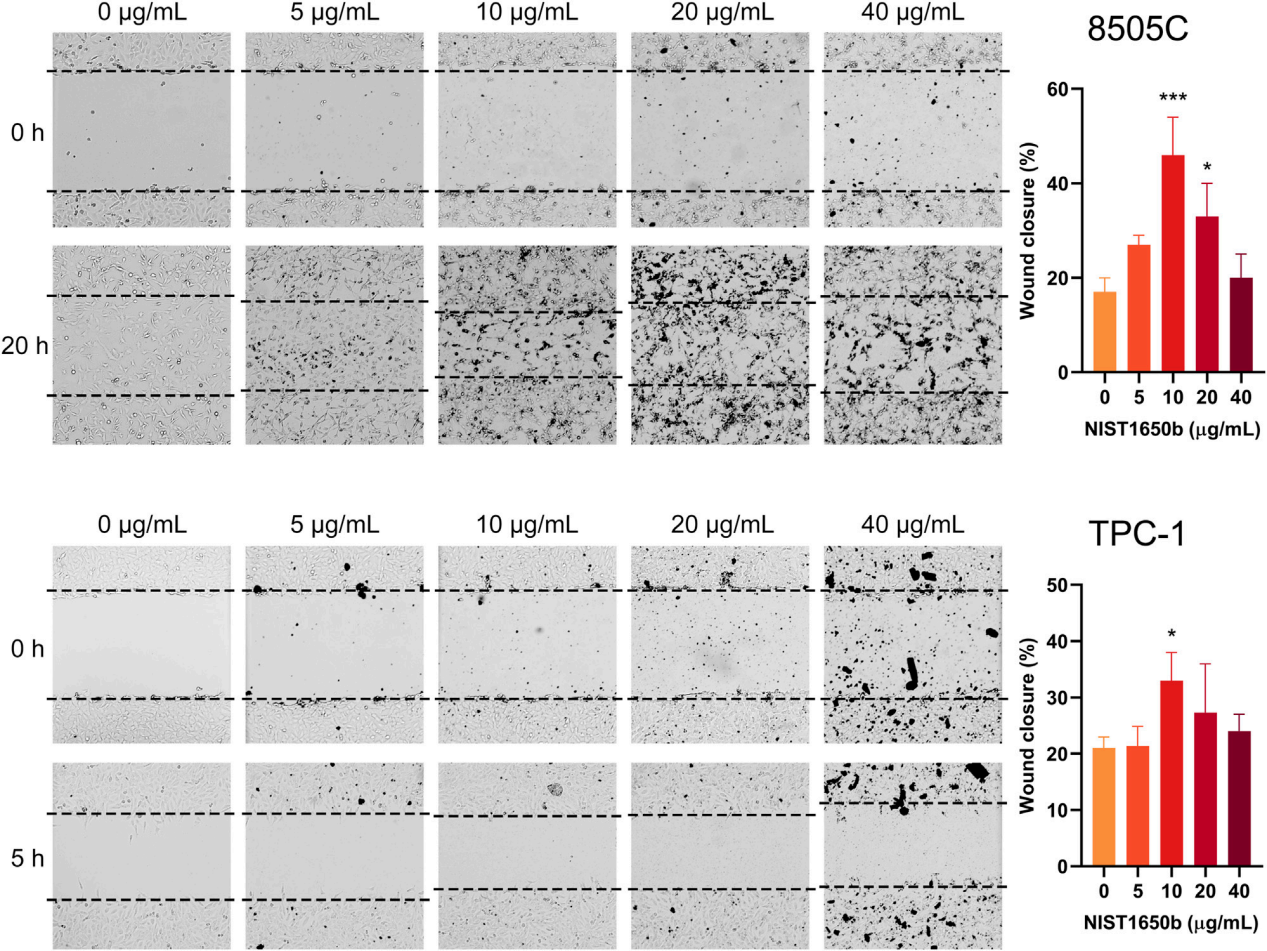
Effects of acute exposure to diesel particulate matter on collective cell migration in thyroid cancer cells. Human thyroid cancer cells 8505C and TPC-1 were treated with different concentrations of NIST1650b for 5 days followed by wound healing assay. ANOVA with Dunnett’s test: *, *p* < 0.05; ***, *p* < 0.001.

Wound healing assays were performed to determine the effects of diesel particulate matter exposure on collective cell migration. Interestingly, we observed that migrating cells in the control group showed cellular disassembly and dispersed detachment from the cluster mass toward wound gaps. NIST1650b-treated cells were held together by cell-cell adhesion, and leading-edge cells formed protruding tips maintaining the leader-follower organization (Figure 3). As such, the leading edge of coherent cells advanced farther than control cells, in which individually migrating cells were dispersedly detached from the coherent group of cells. When we measured the area of the leading edge of coherent cells, 8505C and TPC-1 cells treated with 10 μg/mL NIST1650b had a significantly higher ratio of wound closure than control cells (Figure 4). A significant difference was also seen in 8505C cells treated with 20 μg/mL NIST1650b but not in TPC-1 cells. Taken together, the morphological changes induced by diesel particulate matter were accompanied by augmented collective cell migration in thyroid cancer cells.

**FIGURE 5:**
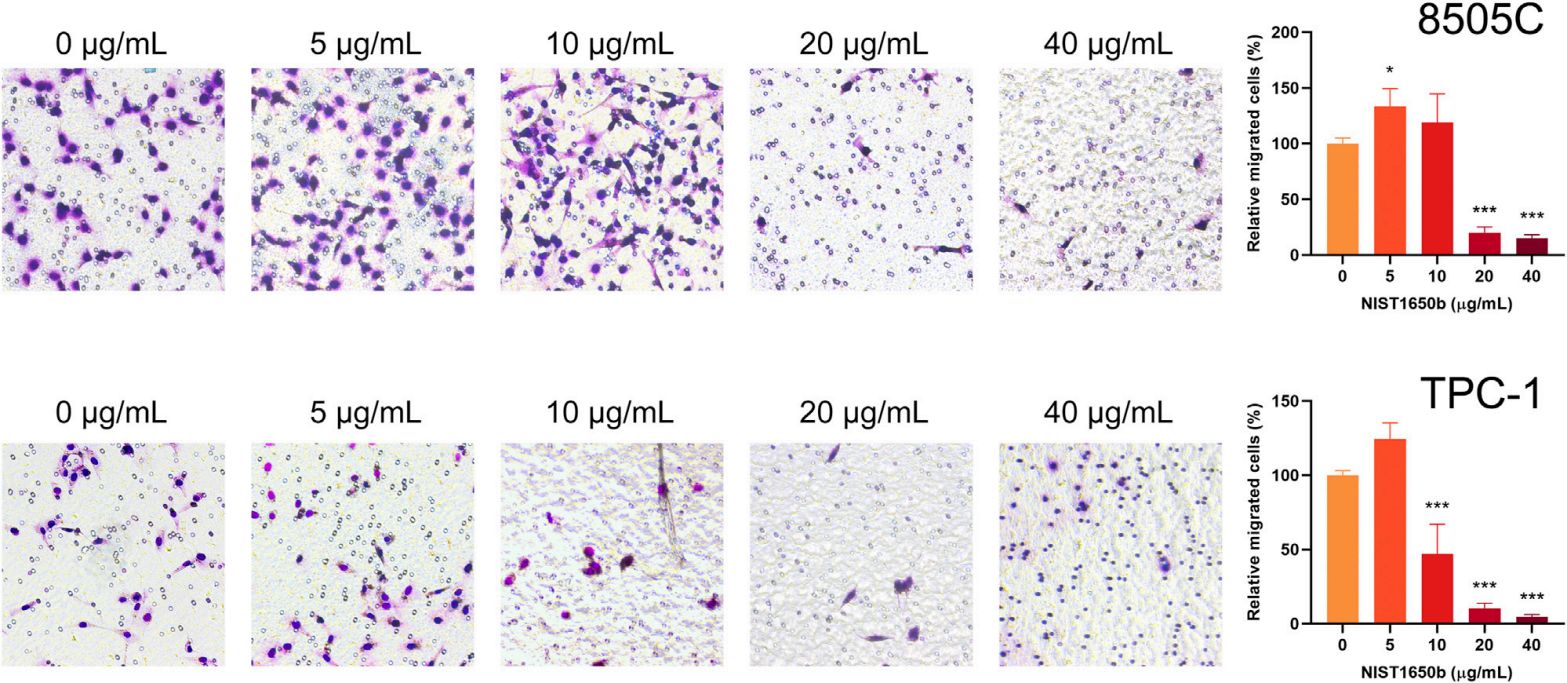
Effects of acute exposure to diesel particulate matter on individual cell migration in thyroid cancer cells. Human thyroid cancer cells 8505C and TPC-1 were treated with different concentrations of NIST1650b for 5 days followed by transwell migration assay. ANOVA with Dunnett’s test: *, *p* < 0.05; ***, *p* < 0.001.

By contrast, the transwell cell migration assay measures the ability of individual cells to move toward a chemoattractant gradient. As shown in Figure 5, a 1.3-fold increase in transwell migration was seen only in 8505C cells treated with 5 μg/mL NIST1650b. TPC-1 cells treated with 5 μg/mL NIST1650b had a marginal 1.2-fold increase in motility without reaching statistical significance (*p* = 0.053). At NIST1650b concentrations of 20 μg/mL or higher, individual cell motility was significantly suppressed in both cell lines. These results suggest that low concentrations of diesel particulate matter exert relatively weak stimulatory effects on single cell migration in thyroid cancer cells.

## Discussion

In this study, using certified diesel particulate matter as a proxy for PM_2.5_, we found that acute exposure to fine particles can change the phenotype of thyroid cancer cells. Our results substantiate some epidemiological data that air pollution may be associated with thyroid cancer. In the European Union, thyroid cancer incidence had a positive association with exposure to elevated PAH levels (Giannoula et al., 2021). A study using the Korean National Health Insurance Service-Health Screening Cohort data disclosed that thyroid cancer was related to several meteorological parameters such as exposure to nitrogen dioxide (Park et al., 2021). In Shanghai, air pollution from industrial waste gas emissions was associated with the incidence of multiple cancer types, including thyroid cancer (Cong, 2018). Concordantly, outdoor air pollution and particulate matter from outdoor air pollution have been classified as carcinogenic to humans by the International Agency for Research on Cancer in 2013.

While some ecological studies analyzed the effects of an overall mixture of air pollution, others specifically focused on the impact of fine particulate matter on thyroid cancer. In a large study involving 407,415 participants from a health examination database, PM_2.5_ exposure increased the risk of endocrine gland cancer with a hazard ratio of 2.13 (Huang et al., 2022). A case-control study utilizing large-volume electronic medical record data from the Johns Hopkins Medical Institution revealed that after multiple adjustments, 3-year cumulative PM_2.5_ exposure was associated with 1.41-fold increased odds of diagnosis of papillary thyroid cancer (Crepeau et al., 2023). These findings suggest a potential link between fine particulate matter and thyroid cancer, though a causal relationship has yet to be inferred. In this context, mechanistic studies on the interplay between PM_2.5_ and thyroid cancer are urgently needed.

Toxicological studies indicate that PM_2.5_-mediated oxidative stress leads to DNA damage and epigenetic modifications that contribute to tumor development (Santibanez-Andrade et al., 2023). Among a variety of cancer hallmarks, PM_2.5_ has been shown to induce epithelial-mesenchymal transition (EMT) in lung epithelial cells (Xu et al., 2019). EMT is a biological program involved in development and wound healing whereby stationary, adherent epithelial cells acquire the ability to boost mobility, invasion, and therapeutic resistance. In a non-small cell lung cancer cell line, either acute or chronic exposure to PM_2.5_ enhanced cell migration and invasion, accompanied by induction of mesenchymal properties (Wei et al., 2017). The tumor-provoking effects of PM_2.5_ were not only seen in lung epithelial cells but also in other cell types. In hepatocellular carcinoma cells, treatment with NIST1650b increased migration and invasion in a dose-dependent manner (Zhang et al., 2017). The mechanisms underlying the progression-promoting effects of PM_2.5_ are partly mediated by the activation of aryl hydrocarbon receptors (AhRs) (Wang et al., 2023). Of interest, AhR has been shown to be upregulated in thyroid cancer samples and functionally favor the acquisition of a mesenchymal phenotype in thyroid cancer cells (Moretti et al., 2020). In this regard, prior to the initiation of the study, we hypothesized that exposure to PM_2.5_ may also induce EMT in thyroid cancer cells.

Unexpectedly, we found that acute exposure to diesel particulate matter did not endorse mesenchymal fate in thyroid cancer cells. Instead, cells were more coherent following treatment and showed an increase in collective cell migration. In such migration, cells move as sheets, strands, clusters or ducts rather than individually. Collectively migrating cells may move more efficiently than if they migrated separately (Friedl and Gilmour, 2009). Although cell migration at the single-cell level has been studied extensively for decades, collective migration has recently been unraveled to be an integral and important behavioral component of neoplastic cells. In thyroid cancer cells, collective cell migration enables tumor cells to cross otherwise unfavorable substrate areas (Lobastova et al., 2017). How fine particulate matter affects these contact-dependent cell perceptions and polarization has not been well studied. In retinal pigment epithelial cells, PM_2.5_ led to changes in cell morphology and cellular aggregate formation, accompanied by increased motility (Lee et al., 2020). These effects were reversed by treatment with a transforming growth factor-β receptor inhibitor or reactive oxygen species scavenger. It is still unknown whether similar mechanisms operate in thyroid cancer cells.

Several limitations of the present study warrant consideration. First, the actual particle size in our NIST1650b preparations was not determined. According to the NIST certificate, the particle-size distribution for NIST1650b varies depending on sonication due to the agglomeration of particles. We did not use a filter to control for particle size, and cellular effects may be greatly affected by exposure to different particle sizes. Second, we did not know whether the concentrations of NIST1650b we used correspond to the range of potential tissue levels. At the present time, there are no data regarding possible levels of ultra-fine particulate matter in the thyroid gland. Experimental studies have shown that translocation across the air-blood barrier and tissue retention strongly depend on particle characteristics such as specific surface area and charge levels (Kreyling et al., 2014). Third, the effect size on wound closure in this study was relatively small, albeit statistically significant compared to the control group. We expect to validate this possible tumor-promoting effect in three-dimensional models, which could more accurately recapitulate *in vivo* conditions. Despite these limitations, we report a novel mechanism by which PM_2.5_ may modulate the process of thyroid tumorigenesis, and our findings may pave the way for a better understanding of the oncogenic roles of fine particulate matter.

## Conclusion

We for the first time demonstrated that acute exposure to diesel particulate matter induces a morphological shift, leads to a coherent phenotype in thyroid cancer cells, and promotes collective cell migration at low concentrations. Additional mechanistic insights into the link between PM_2.5_ and thyroid cancer need to be explored.

## Data Availability

The raw data supporting the conclusions of this article will be made available by the authors, without undue reservation.

## References

[B1] Cheng S. P. Lee J. J. Chang Y. C. Lin C. H. Li Y. S. Liu C. L. (2020). Overexpression of chitinase-3-like protein 1 is associated with structural recurrence in patients with differentiated thyroid cancer. J. Pathol. 252 (2), 114–124. 10.1002/path.5503 32613636

[B2] Cheng S. Y. Hsu Y. C. Cheng S. P. (2023). Trends in thyroid cancer burden in Taiwan over two decades. Cancer Causes Control 34 (6), 553–561. 10.1007/s10552-023-01694-y 37043112 PMC10092943

[B3] Cong X. (2018). Air pollution from industrial waste gas emissions is associated with cancer incidences in Shanghai, China. Environ. Sci. Pollut. Res. Int. 25 (13), 13067–13078. 10.1007/s11356-018-1538-9 29484620

[B4] Crepeau P. Zhang Z. Udyavar R. Morris-Wiseman L. Biswal S. Ramanathan M. Jr (2023). Socioeconomic disparity in the association between fine particulate matter exposure and papillary thyroid cancer. Environ. Health 22 (1), 20. 10.1186/s12940-023-00972-1 36823621 PMC9948306

[B5] Don Porto Carero A. Hoet P. H. Verschaeve L. Schoeters G. Nemery B. (2001). Genotoxic effects of carbon black particles, diesel exhaust particles, and urban air particulates and their extracts on a human alveolar epithelial cell line (A549) and a human monocytic cell line (THP-1). Environ. Mol. Mutagen. 37 (2), 155–163. 10.1002/em.1023 11246222

[B6] Friedl P. Gilmour D. (2009). Collective cell migration in morphogenesis, regeneration and cancer. Nat. Rev. Mol. Cell Biol. 10 (7), 445–457. 10.1038/nrm2720 19546857

[B7] Garcia A. Santa-Helena E. De Falco A. de Paula Ribeiro J. Gioda A. Gioda C. R. (2023). Toxicological effects of fine particulate matter (PM2.5): health risks and associated systemic injuries-systematic review. Water Air Soil Pollut. 234 (6), 346. 10.1007/s11270-023-06278-9 37250231 PMC10208206

[B8] Giannoula E. Melidis C. Frangos S. Papadopoulos N. Koutsouki G. Iakovou I. (2021). Ecological study on thyroid cancer incidence and mortality in association with European Union member states' air pollution. Int. J. Environ. Res. Public Health 18 (1), 153. 10.3390/ijerph18010153 PMC7795041 33379238

[B9] Hsu Y. C. Cheng S. Y. Chien M. N. Cheng S. P. (2023). Impact of social and economic factors on global thyroid cancer incidence and mortality. Eur. Arch. Otorhinolaryngol. 280 (9), 4185–4193. 10.1007/s00405-023-07992-0 37095323

[B10] Huang Y. J. Lee P. H. Chen L. C. Lin B. C. Lin C. Chan T. C. (2022). Relationships among green space, ambient fine particulate matter, and cancer incidence in Taiwan: a 16-year retrospective cohort study. Environ. Res. 212 (Pt C), 113416. 10.1016/j.envres.2022.113416 35523280

[B11] Ishizaka Y. Itoh F. Tahira T. Ikeda I. Ogura T. Sugimura T. (1989). Presence of aberrant transcripts of ret proto-oncogene in a human papillary thyroid carcinoma cell line. Jpn. J. Cancer Res. 80 (12), 1149–1152. 10.1111/j.1349-7006.1989.tb01645.x 2516841 PMC5917935

[B12] Ito T. Seyama T. Iwamoto K. S. Hayashi T. Mizuno T. Tsuyama N. (1993). *In vitro* irradiation is able to cause RET oncogene rearrangement. Cancer Res. 53 (13), 2940–2943. 8319199

[B13] Kreyling W. G. Hirn S. Moller W. Schleh C. Wenk A. Celik G. (2014). Air-blood barrier translocation of tracheally instilled gold nanoparticles inversely depends on particle size. ACS Nano 8 (1), 222–233. 10.1021/nn403256v 24364563 PMC3960853

[B14] Kuo C. Y. Chang Y. C. Chien M. N. Jhuang J. Y. Hsu Y. C. Huang S. Y. (2022). SREBP1 promotes invasive phenotypes by upregulating CYR61/CTGF via the Hippo-YAP pathway. Endocr. Relat. Cancer 29 (2), 47–58. 10.1530/ERC-21-0256 34821220

[B15] Kuo C. Y. Hsu Y. C. Liu C. L. Li Y. S. Chang S. C. Cheng S. P. (2023). SOX4 is a pivotal regulator of tumorigenesis in differentiated thyroid cancer. Mol. Cell. Endocrinol. 578, 112062. 10.1016/j.mce.2023.112062 37673293

[B16] Landa I. Pozdeyev N. Korch C. Marlow L. A. Smallridge R. C. Copland J. A. (2019). Comprehensive genetic characterization of human thyroid cancer cell lines: a validated panel for preclinical studies. Clin. Cancer Res. 25 (10), 3141–3151. 10.1158/1078-0432.CCR-18-2953 30737244 PMC6522280

[B17] Lee H. Hwang-Bo H. Ji S. Y. Kim M. Y. Kim S. Y. Park C. (2020). Diesel particulate matter2.5 promotes epithelial-mesenchymal transition of human retinal pigment epithelial cells via generation of reactive oxygen species. Environ. Pollut. 262, 114301. 10.1016/j.envpol.2020.114301 32155554

[B18] Lim H. Devesa S. S. Sosa J. A. Check D. Kitahara C. M. (2017). Trends in thyroid cancer incidence and mortality in the United States, 1974-2013. JAMA 317 (13), 1338–1348. 10.1001/jama.2017.2719 28362912 PMC8216772

[B19] Liu C. L. Hsu Y. C. Kuo C. Y. Jhuang J. Y. Li Y. S. Cheng S. P. (2022). CRABP2 is associated with thyroid cancer recurrence and promotes invasion via the integrin/FAK/AKT pathway. Endocrinology 163 (12), bqac171. 10.1210/endocr/bqac171 36240291

[B20] Lobastova L. Kraus D. Glassmann A. Khan D. Steinhauser C. Wolff C. (2017). Collective cell migration of thyroid carcinoma cells: a beneficial ability to override unfavourable substrates. Cell. Oncol. 40 (1), 63–76. 10.1007/s13402-016-0305-5 PMC13001555 27826898

[B21] Moretti S. Nucci N. Menicali E. Morelli S. Bini V. Colella R. (2020). The aryl hydrocarbon receptor is expressed in thyroid carcinoma and appears to mediate epithelial-mesenchymal-transition. Cancers 12 (1), 145. 10.3390/cancers12010145 31936153 PMC7016998

[B22] Park S. J. Min C. Yoo D. M. Choi H. G. (2021). National cohort and meteorological data based nested case-control study on the association between air pollution exposure and thyroid cancer. Sci. Rep. 11 (1), 21562. 10.1038/s41598-021-00882-7 34732774 PMC8566463

[B23] Ratajczak M. Gawel D. Godlewska M. (2021). Novel inhibitor-based therapies for thyroid cancer-an update. Int. J. Mol. Sci. 22 (21), 11829. 10.3390/ijms222111829 34769260 PMC8584403

[B24] Sanabria A. Kowalski L. P. Shah J. P. Nixon I. J. Angelos P. Williams M. D. (2018). Growing incidence of thyroid carcinoma in recent years: factors underlying overdiagnosis. Head. Neck 40 (4), 855–866. 10.1002/hed.25029 29206325 PMC5849517

[B25] Santibanez-Andrade M. Quezada-Maldonado E. M. Rivera-Pineda A. Yi C. Garcia-Cuellar C. M. Sanchez-Perez Y. (2023). The Road to malignant cell transformation after particulate matter exposure: from oxidative stress to genotoxicity. Int. J. Mol. Sci. 24 (2), 1782. 10.3390/ijms24021782 36675297 PMC9860989

[B26] Suarez-Arnedo A. Torres Figueroa F. Clavijo C. Arbelaez P. Cruz J. C. Munoz-Camargo C. (2020). An image J plugin for the high throughput image analysis of *in vitro* scratch wound healing assays. PLoS One 15 (7), e0232565. 10.1371/journal.pone.0232565 32722676 PMC7386569

[B27] Wang T. H. Huang K. Y. Chen C. C. Chang Y. H. Chen H. Y. Hsueh C. (2023). PM2.5 promotes lung cancer progression through activation of the AhR-TMPRSS2-IL18 pathway. EMBO Mol. Med. 15 (6), e17014. 10.15252/emmm.202217014 36975376 PMC10245036

[B28] Wei H. Liang F. Cheng W. Zhou R. Wu X. Feng Y. (2017). The mechanisms for lung cancer risk of PM2.5: induction of epithelial-mesenchymal transition and cancer stem cell properties in human non-small cell lung cancer cells. Environ. Toxicol. 32 (11), 2341–2351. 10.1002/tox.22437 28846189

[B29] Xu Z. Ding W. Deng X. (2019). PM2.5, Fine particulate matter: a novel player in the epithelial-mesenchymal transition? Front. Physiol. 10, 1404. 10.3389/fphys.2019.01404 31849690 PMC6896848

[B30] Zhang Q. Luo Q. Yuan X. Chai L. Li D. Liu J. (2017). Atmospheric particulate matter2.5 promotes the migration and invasion of hepatocellular carcinoma cells. Oncol. Lett. 13 (5), 3445–3450. 10.3892/ol.2017.5947 28521450 PMC5431175

